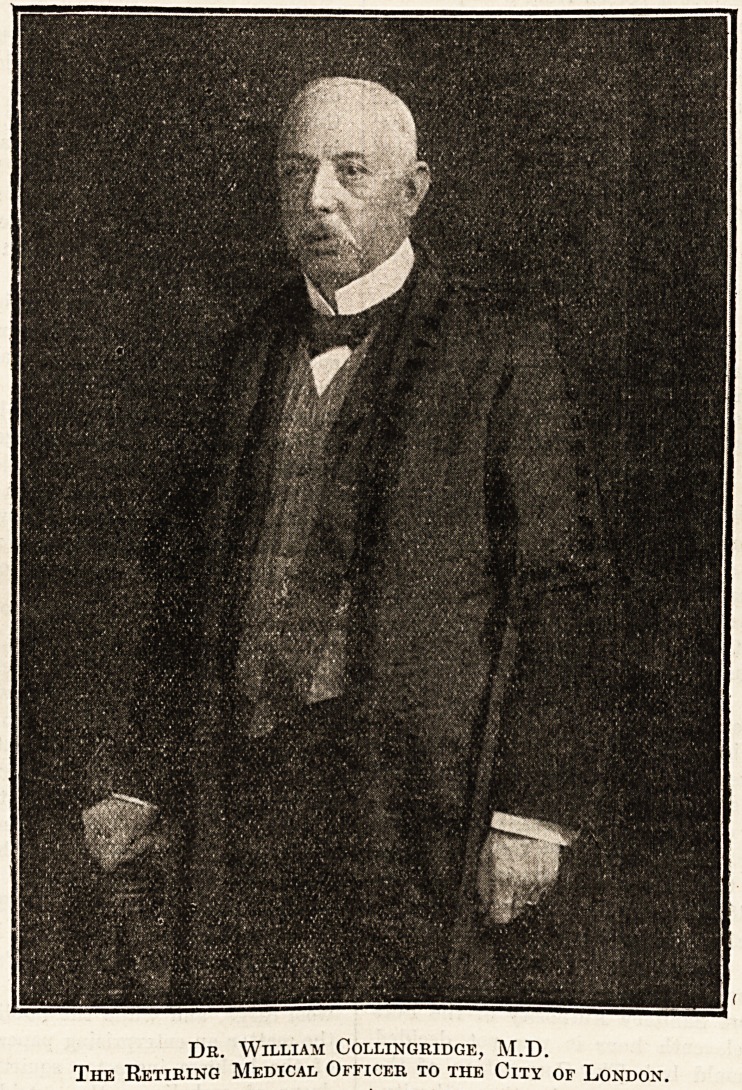# Thirty-Two Years of Public Health

**Published:** 1911-12-16

**Authors:** 


### THIRTY-TWO YEARS OF PUBLIC HEALTH.

### Interview with Dr. W. Collingridge on his Retirement.

### I.—AT THE PORT OF LONDON.

Very soon after Dr. William Collingridge's retirement from the post of Medical Officer of Health for the City was announced, he consented very courteously to give our Commissioner a synopsis of the changes that have taken place during the thirty-two years in which he has been connected with public health. Remembering that Dr. Collingridge was appointed Medical Officer to the Port of London in 1880, I asked him first how he became interested in public preventive medicine?

"I was doing very well at private practice," he rejoined, "but six months after I started, the post of Medical Officer to the Port of London fell vacant. My friends suggested that I should apply for it, but though I was successful the post was not actually filled for eighteen months. So altogether I had two years at private practice."

A Nelson Relic.

"Was the work very heavy at that time?"

"No, it was small. We had one inspector and one launch, and the only hospital ship was the old *Rhin*, which had been taken by Nelson in 1804, so you can imagine how old - fashioned our appliances were."

"These deficiencies were brought into prominence by the cholera?"

"Yes; that broke out, I think, in 1884 at Hamburg. The Customs officials were completely beaten, and eventually a sea-going vessel was procured, and a hospital was built on shore, at Denton, near Gravesend. That gave us twenty-four beds. But this was not enough. A system of medical inspection on ships also was inaugurated."

Dr. William Collingridge, M.D.
The Retiring Medical Officer to the City of London.

"The cholera then produced some good results?"

"Yes; all reforms date from epidemics. When the second cholera epidemic broke out in 1892, again in Hamburg, still more important results were obtained. Quarantine was disregarded. I regard that as a most important step; and in its place medical inspection of every vessel coming from an infected port was undertaken. The Corporation gave me a free hand, and no vessel might be cleared without my sanction. Moreover an isolation camp was built in the grounds of the hospital at Gravesend. There was, you see, no quarantine station nearer than the old one at Standgate Creek. When quarantine was disregarded the Local Government Board, which in those days had cognisance of some matters affecting public health, gave a tardy sanction to what had been done, which, of course, strictly speaking, was in defiance of the regulations."

"Why were you opposed to quarantine?"

"Quarantine simply meant the detention of a vessel which came from an infected port, without any regard to the actual health of the crew or of the passengers. Medical inspection, on the other hand, disregards the fact that the port of lading is infected, except in so far as it provides a reason for the inspection of the passengers and the crew."

"From the passengers' point of view, the change was an immense improvement?"

"Of course, instead of being forbidden to land, their addresses merely were taken, and they had to give an undertaking to report themselves at a given interval to the local medical officer to whose district they were going."

"Why, then, to digress, can't quarantine for dogs be done away with?"

"It depends on the nature of the disease. You must understand that the incubation period of cholera is only five days. That of rabies about as many months."

"Did the Customs officials agree willingly to the innovation?"

"Not very willingly," said Dr. Collingridge, smiling at the recollection of "old, unhappy, far-off things, and battles long ago."

"There were," he went on, "as a matter of fact, three stages in our fight with them. First of all we forced our inspector on their hulk, a proceeding which encountered not merely moral opposition! Then the Corporation got a hulk of its own, and finally we launched our medical inspectors."

"How many are there to-day?"

Dr. Collingridge turned up a report. "There are four medical officers now always on duty at Gravesend and one at Sheerness. Sometimes it is hard, but there are compensations. The hours are twenty-four on and forty-eight off. That sounds rather worse than it is, for the inspections can be made only at the flow tide, and fog and other weather conditions interfere with the regularity of the work."

"And besides medical inspection . . . ?"

"Well, the next thing was to secure the notification of diseases other than quarantine diseases—other, that is, than cholera, yellow fever, and plague. The Public Health Act of 1875 gave one certain powers, but what was wanted was power to remove a person from a ship in the same way that a person could be removed from a house."

"When was quarantine finally abolished?"

"In 1896, by the Quarantine or Ships Act. I forget the exact title. Medical inspection was then substituted, and now there are three launches and the whole river is patrolled. Now, just compare the efficiency of this system with the condition of affairs so recent as in 1892. Then, if you please, the Custom House officials could not get lodgings in Gravesend for fear of the cholera! My successor at the Port of London was Dr. Williams Herbert, who was appointed in 1901, and he has been carrying on the new system so well that to-day in many respects it could hardly be more efficient than it now is. Questions that were only beginning to agitate my department have now come into great prominence. For instance, take the case of plague and the destruction of rats. That alone will give you some idea of the way in which the work has developed since my departure."

### The Brightest Jewel in the City's Crown.

"Perhaps it is late to ask, but what is exactly meant by the Port of London?"

"The Port stretches from Teddington to the mouth of the Medway. It is divided into sections, of which the docks form one. One sanitary inspector visits each dock each day, and such inspection dates in its earliest form from 1880."

"And your part in encouraging this?"

"Oh, my part has been, as I used to sum it up to the Corporation, to regard ships as houses afloat, and houses as if they were ships on shore!"

"Have you anything else to emphasise about the Port?"

"There is," said Dr. Collingridge with interest, "one little piece of history that is certainly worth recording, and that is this. In 1872 the Public Health Act, which constituted the authority of the Port of London, got actually as far as the Committee stage in the House of Commons with a clause containing these words: 'The . . . . shall be the Port Sanitary Authority of the Port of London.' At the eleventh hour it was not decided who that authority should be! The Corporation of the City came forward and undertook to be the authority, and were allowed to do so on condition that they should pay all expenses out of their Corporate funds. They do so still, and we have this wonderful fact, that the Port of London is absolutely no burden on the ratepayers."

"What is its annual cost?"

"Now it is about £10,000 a year. In my time the expenses, I am glad to confess, jumped up from about three to seven thousand."

"Has the Corporation ever regretted its undertaking?"

"Never! Why, more than one Lord Mayor has described it as the brightest jewel in the Corporation's crown, and all the rest of it! No," said Dr. Collingridge more quietly, "it has brought immense credit to the Corporation."

### Frozen Meat and its Problems.

A pause here followed, for in the interest that we were taking in the story of modern sanitation as applied to the Port of London with its quaint side-lights on a former state of things, above all with Dr. Collingridge's reminder that all this was brought about informally without any powers, till Parliament sanctioned these experiments in progress, I had almost forgotten that Dr. Collingridge had promised to relate something of his work as Medical Officer to the City.

"What is the greatest improvement of recent years?" I asked.

"The carrying through of the proper inspection of food," said Dr. Collingridge slowly. "I will give you one instance of the increase and the value of the work in this respect. In 1880 there was no frozen meat imported into this country. Think what a change that means."

"You recall that innovation?"

"Yes, I recollect quite well seeing the first cargo of frozen meat come in. Now we have specially qualified food inspectors, and a specimen out of each ship's cargo is examined by them."

"How are their duties comprised?"

"You can tell that from their qualifications. Each inspector has to hold a master mariner's certificate, as well as that of the Royal Sanitary Institute. The inspector must hold the former to give him a standing with the masters of ships. The knowledge that implies will prevent a man from insisting on alterations that no ship's master could possibly carry out. Then the Board of Trade had certain jurisdiction (as it has now, of course, over matters of construction), over sanitary matters, but now always gives way to the recommendations of the Port."

### The Chance of 1897.

"I should have said earlier," remarked Dr. Collingridge, "that in my fight against quarantine I had one great opportunity. That was in 1897, when I was appointed Milroy Lecturer to the Royal College of Physicians. You can guess what subject I took, 'Quarantine'! I was able then to marshal my case against it, and to attract attention in that way."

"Did you use any other means?"

"Well, as a matter of fact the case against quarantine was very strikingly illustrated. In about 1894, I think it was, the *Arawa* came into the Thames with yellow fever among her crew. I would not have anything to do with the case, and left it for the Privy Council to carry out the ritual of disinfection then in vogue. She carried a wool cargo, and while the public interest was excited in the matter an enterprising paper published an illustration of the luckless inspector squirting with a little syringe drops of carbolic on the outside of the carcases, which were heavily encased in sacking! The result was that a roar of laughter went up, and these methods of disinfecting were killed by ridicule. Ridicule and epidemics are two very good ways of getting reforms!"

---

Next week we shall publish Dr. Collingridge's résumé of the work that now falls to the Chief Medical Officer of London, and how that work has developed to its present extent.

## Figures and Tables

**Figure f1:**